# Supplementary material for: Complexome profiling of the Chlamydomonas psb28 mutant reveals TEF5 as an early PSII assembly factor
Source: Plant Cell. 2025 Mar 17;37(6):koaf055. doi: 10.1093/plcell/koaf055 (PMC12164590; doi:10.1093/plcell/koaf055)
Supplement: koaf055_Supplementary_Data [file koaf055_supplementary_data.zip › psb28_interactive.zip!/plots/intensity/qualityPlots/peptides/_Cre03.g182050.t1.2.html]

### Cre03.g182050.t1.2

Trivial name:   
SeqCoverage [median]: 20.70  
Mass: 72.25 kDa  
  
 

| Sequence | MissedCleavage | Mass [Da] | wtIntensity | muIntensity | Score |
| --- | --- | --- | --- | --- | --- |
| AAVLAAMTATGK | 0 | 1103.60 | 2711.81 | 5009.98 | 142.00 |
| ALASAPVK | 0 | 755.45 | 475.95 | 1107.94 | 69.18 |
| LNSLEQVK | 0 | 929.52 | 2973.25 | 0.00 | 111.01 |
| LSQGEYIAVEK | 0 | 1235.64 | 2462.28 | 2203.78 | 121.60 |
| LVVSAGGK | 0 | 729.44 | 904.09 | 1658.81 | 91.91 |
| QVDAMYAELAEAEAAK | 0 | 1708.80 | 0.00 | 674.14 | 110.38 |
| VLSWEELVTTGK | 0 | 1360.72 | 410.91 | 0.00 | 94.30 |
| VLVSGSAPLSQQMESFMR | 0 | 1965.97 | 1390.98 | 1736.85 | 57.52 |
|  |  |  | 11329.3 | 12391.5 |  |
